# TRAUMA, PERSONALITY, AND DEPRESSION AMONG OLDER ADULTS DURING THE COVID-19 PANDEMIC

**DOI:** 10.1093/geroni/igad104.1958

**Published:** 2023-12-21

**Authors:** Alejandra Zamora, Richard Zweig

**Affiliations:** Dartmouth College , Lebanon, New Hampshire, United States; Ferkauf Graduate School of Psychology, Yeshiva University, Bronx, New York, United States

## Abstract

Older adults who have experienced early trauma are predisposed to develop mental health problems such as depression, though further evidence is needed to examine the roles of personality traits and interpersonal problems. The current study examined the respective influences of personality traits and interpersonal problems in the relationship between early trauma and depressive symptoms among middle and older adults who had experienced a loss during the COVID-19 pandemic (N=214). Participants were 76% female and ranged in age from 55-84 (Age M= 62.7, SD= 5.89). Twelve participants who endorsed at least adverse childhood experience were interviewed regarding their childhood and pandemic experiences. Categorical analyses found significant differences between individuals with high and low levels of ACEs with regard to neuroticism (d=-.32), depression (d=-.28), and interpersonal problems (d=-.55). High neuroticism and low extraversion were significantly predictive of interpersonal problems (ß= .520, p< .001; ß=-.201, p= .004) and depression (ß = .588, p< .001; ß= -.177, p= .006). Early trauma and interpersonal problems were not predictive of depression after accounting for variance due to personality traits. Moderation analyses were non-significant. Qualitative analyses yielded themes of re-encountering problems faced as children and adaptation or perseverance of strategy. These findings suggest that personality appeared to contribute significantly to depressive symptoms among bereaved older adults during the COVID-19 pandemic. Early trauma may influence depression in later life with regard to ease of adaptation to contexts and challenges.

